# Policy implications of mandatory systemic immunosuppressant trials prior to dupilumab for pediatric atopic dermatitis in Taiwan

**DOI:** 10.3389/fped.2026.1767209

**Published:** 2026-03-31

**Authors:** Hao-Yun Chen, Jhe-Cyun Liao, Po-Cheng Shih, Su-Boon Yong, Chia-Jung Li

**Affiliations:** 1 School of Medicine, China Medical University, Taichung, Taiwan; 2 Division of Allergy, Immunology, Rheumatology, Changhua Christian Hospital, Changhua, Taiwan; 3 Institute of Medicine, Chung Shan Medical University, Taichung, Taiwan; 4 Department of Allergy and Immunology, China Medical University Children’s Hospital, Taichung, Taiwan; 5 Research Center for Allergy, Immunology, and Microbiome (A.I.M.), China Medical University Hospital, Taichung, Taiwan; 6 Department of Obstetrics and Gynecology, Kaohsiung Veterans General Hospital, Kaohsiung, Taiwan; 7 Institute of Biopharmaceutical Sciences, National Sun Yatsen University, Kaohsiung, Taiwan

**Keywords:** atopic dermatitis, biologic therapy, dupilumab, health policy, methotrexate, pediatric dermatology, taiwan

## Abstract

The increasing use of dupilumab for moderate-to-severe atopic dermatitis (AD) in Taiwan has drawn attention to the country's reimbursement policy requiring a mandatory three-month trial of systemic immunosuppressants such as methotrexate, azathioprine, or cyclosporine before approval of biologic therapy in children aged 6–12 years. This stepwise approach aims to optimize healthcare resource allocation and ensure that biologics are reserved for refractory cases. However, concerns have been raised regarding potential delays in effective disease control, prolonged disease burden, and exposure to adverse effects associated with traditional immunosuppressants, including hepatotoxicity and infection risk. In contrast, Japan allows direct access to dupilumab without prior systemic therapy, emphasizing early intervention and improved long-term outcomes, though patients must bear 30% of treatment costs. This policy divergence between Taiwan and Japan underscores broader challenges in balancing cost-effectiveness, clinical efficacy, and equity in healthcare delivery. Ongoing evaluation of reimbursement criteria will be essential to align national policies with evolving therapeutic evidence and patient-centered care goals.

The introduction of dupilumab, a biologic medication approved for moderate-to-severe atopic dermatitis (AD), has significantly reshaped the therapeutic landscape for pediatric dermatology in Taiwan. Following the implementation of a national reimbursement policy that subsidizes dupilumab, its clinical utilization has risen notably among pediatric populations. However, eligibility for reimbursement is contingent upon completing a mandatory three-month course of conventional systemic immunosuppressants—methotrexate, azathioprine, or cyclosporine—administered at standard therapeutic doses and durations, unless contraindicated due to intolerance or adverse reactions (1).

This prerequisite has sparked considerable debate among clinicians, patient advocacy groups, and policymakers. To contextualize the scope of this issue, nationwide epidemiological studies using Taiwan's National Health Insurance database reveal that the prevalence of atopic dermatitis in pediatric populations is approximately 9.6%–10%, with an estimated 20%–25% of these children suffering from moderate-to-severe disease that frequently requires systemic intervention (2). Critics argue that enforcing this mandatory trial of conventional drugs may unnecessarily prolong disease burden in these vulnerable pediatric patients, especially when disease control remains suboptimal. Adverse effects such as gastrointestinal distress, hepatotoxicity, and increased infection risk may further compromise adherence and quality of life (3, 4). Furthermore, delayed initiation of biologic therapy could result in preventable disease progression and chronic skin barrier dysfunction, both of which contribute to long-term psychosocial impacts. From an immunological perspective, pediatric AD is primarily driven by systemic Type 2 inflammation, specifically mediated by interleukins (IL)-4 and IL-13. These cytokines not only provoke chronic cutaneous inflammation and pruritus but also critically impair epidermal barrier function (5). Importantly, early-onset AD frequently acts as the starting point for the ‘atopic march'—a progressive trajectory from eczema to subsequent IgE-mediated comorbidities, including food allergies, asthma, and allergic rhinitis (6). Emerging literature demonstrates that early, targeted blockade of IL-4Rα with dupilumab can effectively control this systemic Type 2 inflammation, restore skin barrier integrity, and potentially alter the long-term disease course to mitigate the risk of the atopic march (5, 7). In contrast, conventional systemic immunosuppressants, while broadly dampening immune responses, fail to specifically target this core pathogenic pathway. Delaying biologic therapy by mandating traditional immunosuppressants may thus cause pediatric patients to miss the critical window for true disease modification (5).

Conversely, proponents defend the current policy as a pragmatic strategy for optimizing limited healthcare resources. The economic disparity between the treatments is stark: the annual cost of dupilumab therapy in Taiwan is approximately NT$ 330,000–NT$ 350,000 per patient, compared to roughly NT$ 5,000–NT$ 10,000 for conventional immunosuppressants (8). Given this substantial cost difference, a stepwise treatment framework encourages rational prescribing and cost containment. This ensures that high-cost biologics are reserved for patients with refractory or treatment-resistant disease, maintaining sustainability within the National Health Insurance system (9). Such policies reflect Taiwan's broader efforts to balance innovation, access, and affordability within a single-payer healthcare system.

In contrast, Japan has implemented a more direct-access approach to dupilumab, without requiring prior immunosuppressive therapy in pediatric AD patients. The rationale emphasizes early disease control, reduced exposure to systemic drug toxicity, and improved adherence (10). However, Japan's National Health Insurance covers only 70% of the cost, leaving families responsible for the remaining 30%, which may create a substantial financial barrier for some households. Thus, while Taiwan's model prioritizes system sustainability, Japan's approach prioritizes early intervention and patient quality of life—two distinct but equally valid public health strategies. To contextualize the Taiwan-Japan dichotomy, it is instructive to examine broader international paradigms. Globally, reimbursement frameworks for dupilumab in pediatric AD exist on a spectrum between strict cost-containment and early biological intervention. For instance, the United Kingdom's National Institute for Health and Care Excellence (NICE) guidelines adopt a step-therapy approach akin to Taiwan's, requiring patients to demonstrate an inadequate response to, or intolerance of, at least one conventional systemic immunosuppressant (e.g., cyclosporine or methotrexate) prior to dupilumab approval (11). Conversely, in the United States, while private insurance mandates vary significantly, recent updates to dermatological consensus guidelines increasingly advocate for direct biologic access in moderate-to-severe pediatric cases, bypassing traditional immunosuppressants due to their unfavorable long-term safety profiles (12). By framing Taiwan's policy within this global context, it becomes evident that the ongoing debate is not unique to Taiwan, but reflects a universal healthcare challenge: reconciling the high upfront costs of targeted biologics with the long-term clinical imperatives of pediatric disease modification.

These contrasting policies between Taiwan and Japan highlight a broader global dilemma in the management of chronic dermatologic conditions: how to balance treatment efficacy, safety, and equity against the realities of economic sustainability. As biologic therapies continue to redefine chronic disease management, regular policy reassessment will be critical to ensure that access criteria evolve in tandem with clinical evidence and patient-centered care principles.

## References

[1] National Health Insurance Administration, Ministry of Health and Welfare. Section 13: Dermatological Preparations—latest Version of the Drug Reimbursement Criteria (2023). Taipei city: National Health Insurance Administration, Ministry of Health and Welfare. Chinese. Available online at: https://www.nhi.gov.tw/ch/dl-66483-865569bb4e6047e792e8481766710b30-1.pdf (Accessed October 21, 2025).

[2] Hwang CY Chen YJ Lin MW Chen TJ Chu SY Chen CC Prevalence of atopic dermatitis, allergic rhinitis and asthma in Taiwan: a national study 2000 to 2007. Acta Derm Venereol. (2010) 90:589–94. 10.2340/00015555-0963 21057741

[3] Elsgaard S Danielsen AK Thyssen JP Deleuran M Vestergaard C. Drug survival of systemic immunosuppressive treatments for atopic dermatitis in a long-term pediatric cohort. Int J Womens Dermatol. (2021) 7(5 Pt B):708–15. 10.1016/j.ijwd.2021.07.005 35028369 PMC8714597

[4] Schram ME Roekevisch E Leeflang MM Bos JD Schmitt J Spuls PI. A randomized trial of methotrexate versus azathioprine for severe atopic eczema. J Allergy Clin Immunol. (2011) 128(2):353–9. 10.1016/j.jaci.2011.03.024 21514637

[5] Kim JH Samra MS. Moderate to severe atopic dermatitis in children: focus on systemic Th2 cytokine receptor antagonists and Janus kinase inhibitors. Clin Exp Pediatr. (2024) 67(2):64–79. 10.3345/cep.2022.00346 37321570 PMC10839191

[6] Davidson WF Leung DYM Beck LA Berin CM Boguniewicz M Busse WW Report from the national institute of allergy and infectious diseases workshop on “atopic dermatitis and the atopic march: mechanisms and interventions”. J Allergy Clin Immunol. (2019) 143(3):894–913. 10.1016/j.jaci.2019.01.003 30639346 PMC6905466

[7] Lin TL Fan YH Fan KS Juan CK Chen YJ Wu CY. Reduced atopic march risk in pediatric atopic dermatitis patients prescribed dupilumab versus conventional immunomodulatory therapy: a population-based cohort study. J Am Acad Dermatol. (2024) 91(3):466–73. 10.1016/j.jaad.2024.05.029 38878041

[8] Lee EM Cho YT Chan TC Shen D Chu CY Tang CH. Economic burden of atopic dermatitis in Taiwan. Acta Derm Venereol. (2023) 103:adv00869. 10.2340/actadv.v103.4556 36789754 PMC9944199

[9] Lee EM Cho YT Hsieh WT Chan TC Shen D Chu CY Healthcare utilization and costs of atopic dermatitis in Taiwan. J Formos Med Assoc. (2022) 121(10):1963–71. 10.1016/j.jfma.2022.01.028 35177295

[10] de Wijs LEM Fujimoto RFT Andrinopoulou ER Nijsten T Hijnen D Kataoka Y. Dupilumab treatment in patients with atopic dermatitis: a comparative cohort study between The Netherlands and Japan shows a discrepancy in patient-reported outcome measures. Br J Dermatol. (2021) 185(3):555–62. 10.1111/bjd.19897 33657668 PMC8453550

[11] National Institute for Health and Care Excellence (NICE). Dupilumab for Treating Moderate to Severe Atopic Dermatitis in Children Aged 6 to 11 Years (Technology Appraisal Guidance [TA751]). London: NICE (2021).

[12] Boguniewicz M Alexis AF Beck LA Block J Eichenfield LF Fonacier L Expert perspectives on management of moderate-to-severe atopic dermatitis: a multidisciplinary consensus addressing current and emerging therapies. J Allergy Clin Immunol Pract. (2017) 5(6):1519–31. 10.1016/j.jaip.2017.08.005 28970084

